# Novel avenues of tau research

**DOI:** 10.1002/alz.13533

**Published:** 2024-01-03

**Authors:** Claire E. Sexton, Gal Bitan, Kathryn R. Bowles, Miroslaw Brys, Luc Buée, Mahmoud Bukar Maina, Claire D. Clelland, Ann D. Cohen, John F. Crary, Jeffrey L. Dage, Kristophe Diaz, Bess Frost, Li Gan, Alison M Goate, Lawrence I. Golbe, Oskar Hansson, Celeste M. Karch, Hartmuth C. Kolb, Renaud La Joie, Suzee E. Lee, Diana Matallana, Bruce L. Miller, Chiadi U. Onyike, Yakeel T. Quiroz, Jessica E. Rexach, Jonathan D. Rohrer, Amy Rommel, Ghazaleh Sadri‐Vakili, Suzanne E. Schindler, Julie A. Schneider, Reisa A. Sperling, Charlotte E. Teunissen, Stacie C. Weninger, Susan L. Worley, Hui Zheng, Maria C. Carrillo

**Affiliations:** ^1^ Alzheimer's Association Chicago Illinois USA; ^2^ Department of Neurology David Geffen School of Medicine Brain Research Institute Molecular Biology Institute University of California Los Angeles (UCLA) Los Angeles California USA; ^3^ UK Dementia Research Institute at the University of Edinburgh Centre for Discovery Brain Sciences University of Edinburgh Edinburgh UK; ^4^ Eli Lilly & Co. Indianapolis Indiana USA; ^5^ Univ Lille Inserm CHU‐Lille Lille Neuroscience and Cognition LabEx DISTALZ Place de Verdun Lille France; ^6^ Sussex Neuroscience School of Life Sciences University of Sussex Falmer UK; ^7^ Biomedical Science Research and Training Centre Yobe State University Damaturu Nigeria; ^8^ Memory and Aging Center Department of Neurology Weill Institute for Neurosciences University of California San Francisco California USA; ^9^ University of Pittsburgh School of Medicine Department of Psychiatry and Alzheimer's disease Research Center Pittsburgh Pennsylvania USA; ^10^ Departments of Pathology Neuroscience, and Artificial Intelligence & Human Health Icahn School of Medicine at Mount Sinai New York New York USA; ^11^ Department of Neurology Indiana University School of Medicine Indianapolis Indiana USA; ^12^ CurePSP Inc New York New York USA; ^13^ Sam & Ann Barshop Institute for Longevity & Aging Studies Glenn Biggs Institute for Alzheimer's & Neurodegenerative Disorders Department of Cell Systems and Anatomy University of Texas Health San Antonio San Antonio Texas USA; ^14^ Helen and Robert Appel Alzheimer Disease Research Institute Feil Family Brain and Mind Research Institute Weill Cornell Medicine New York New York USA; ^15^ Department of Genetics & Genomic Sciences Ronald M. Loeb Center for Alzheimer's disease Icahn School of Medicine at Mount Sinai New York New York USA; ^16^ Rutgers Robert Wood Johnson Medical School New Brunswick New Jersey USA; ^17^ Clinical Memory Research Unit Department of Clinical Sciences Malmö Lund University Lund Sweden; ^18^ Department of Psychiatry Washington University in St. Louis St. Louis Missouri USA; ^19^ Janssen R&D Neuroscience San Diego California USA; ^20^ Aging Institute Neuroscience Program Psychiatry Department School of Medicine Pontificia Universidad Javeriana Bogotá Colombia; ^21^ Mental Health Department Hospital Universitario Fundaciòn Santa Fe Bogota Colombia; ^22^ Department of Psychiatry and Behavioral Sciences University of California San Francisco California USA; ^23^ Division of Geriatric Psychiatry and Neuropsychiatry Johns Hopkins University School of Medicine Baltimore Baltimore Maryland USA; ^24^ Departments of Psychiatry and Neurology Massachusetts General Hospital Harvard Medical School Boston Massachusetts USA; ^25^ Program in Neurogenetics Department of Neurology David Geffen School of Medicine University of California Los Angeles California USA; ^26^ Department of Neurodegenerative Disease Dementia Research Centre University College London Institute of Neurology, Queen Square London UK; ^27^ Rainwater Charitable Foundation Fort Worth Texas USA; ^28^ Sean M. Healey &AMG Center for ALS at Mass General Massachusetts General Hospital Boston Massachusetts USA; ^29^ Department of Neurology Washington University School of Medicine St. Louis Missouri USA; ^30^ Rush Alzheimer's Disease Center Chicago Illinois USA; ^31^ Center for Alzheimer Research and Treatment Brigham and Women's Hospital Massachusetts General Hospital Harvard Medical School Boston Massachusetts USA; ^32^ Neurochemistry Laboratory Clinical Chemistry department Amsterdam Neuroscience Program Neurodegeneration Amsterdam University Medical Centers Vrije Universiteit Amsterdam Amsterdam The Netherlands; ^33^ FBRI Cambridge Massachusetts USA; ^34^ Independent Science Writer Bryn Mawr Pennsylvania USA; ^35^ Huffington Center on Aging Baylor College of Medicine Houston Texas USA

**Keywords:** biomarkers, tau, tau‐PET, tauopathies, therapeutics

## Abstract

**INTRODUCTION:**

The pace of innovation has accelerated in virtually every area of tau research in just the past few years.

**METHODS:**

In February 2022, leading international tau experts convened to share selected highlights of this work during Tau 2022, the second international tau conference co‐organized and co‐sponsored by the Alzheimer's Association, CurePSP, and the Rainwater Charitable Foundation.

**RESULTS:**

Representing academia, industry, and the philanthropic sector, presenters joined more than 1700 registered attendees from 59 countries, spanning six continents, to share recent advances and exciting new directions in tau research.

**DISCUSSION:**

The virtual meeting provided an opportunity to foster cross‐sector collaboration and partnerships as well as a forum for updating colleagues on research‐advancing tools and programs that are steadily moving the field forward.

## 1 INTRODUCTION

The protein tau has been implicated in the pathogenesis of a number of brain disorders, ranging from tauopathies such as progressive supranuclear palsy (PSP), corticobasal degeneration (CBD), and Alzheimer's disease (AD) to conditions not classically categorized as tauopathies, such as Parkinson's disease, Down syndrome, autism, and epilepsy. Although in recent years innovative and informative disease models and technologies have emerged, many of the primary functions of tau in both disease and physiology, have yet to be clearly defined in each of these disorders. Among significant obstacles to the development of effective therapeutic strategies is the lack of a refined understanding of the signaling pathways that are relevant to diverse biological and pathological processes in each of the tauopathies, as well as their regulation. Research increasingly suggests that tau may serve different functions in different cell types and subcellular locations, under varying circumstances in different diseases, and therefore may participate in a wide range of pathways and mechanisms that can contribute to cell dysfunction and loss. Among important aims moving forward will be to identify these mechanisms and pathways and to establish their relative importance in increasingly informative disease‐specific models.

### 1.1 Biology

Among the many important new research directions discussed at Tau 2020 were those involving a deeper exploration of the fundamental biology of tau.^1^ A growing recognition that the early and nearly exclusive classification of tau as a microtubule‐binding protein likely delayed the investigation of other important, non‐canonical biologic functions of tau^2^ has spurred deeper explorations of tau's structural diversity, its surprising range of posttranslational modifications (as well as their implications), and tau's previously unanticipated presence in a wide range of subcellular locations in both neuronal and non‐neuronal cells. These efforts have involved reexamining and, in some cases, relinquishing long‐held beliefs about the role of tau under both physiological and pathological conditions. Recent studies involving experimental genetic ablation or reduction of tau, for example, have resulted in an absence of detectable changes in the axonal transport of tau or in the stabilization of microtubules.^3^ Tau reduction also does not appear to interfere with biological processes that likely depend on microtubules.^3^ The lack of compelling evidence that tau reduction causes derangements of microtubules or alterations in microtubule dynamics that affect neuronal function or integrity raises questions about earlier assumptions regarding the pathophysiology of AD and other tauopathies – particularly with regard to the primary/pivotal role of tau's detachment from microtubules and subsequent self‐aggregation.

More recent research demonstrating that the internalization of propagating tau can occur without impairing neuronal integrity likewise calls for a reexamination of beliefs about the role of tau propagation during disease.^3^ Moreover, unanticipated findings regarding tau's involvement in pathomechanisms that are *unique* to several different tau‐related disorders have suggested that diverse therapeutic strategies will be required for the treatment of various tauopathies.^3^ Collectively these findings point to multifaceted and multifunctional qualities of tau and suggest that our understanding of its roles in neuronal physiology and pathology may need to be revised in response to novel discoveries.

### 1.2 Normal physiological spread of tau

Most early research on tau spread focused on the cell‐to‐cell transfer of misfolded, abnormal tau in the context of neurodegenerative disease and ultimately led to the proposal that disease likely spreads from diseased to healthy neurons in a characteristic spatial and temporal progression that is mediated by extracellular, abnormal forms of tau.^4^, ^5^ However, more recent research has provided strong evidence of regulated release of non‐pathogenic forms of tau from healthy neurons, as well as non‐pathogenic entry of tau into both neurons and non‐neuronal cells,^6^, ^7^, ^8^, ^9^ and a role for extracellular vesicles in tau spreading.^10^, ^11^, ^12^, ^13^, ^14^ Findings from these studies have suggested that the transfer of tau between neurons might be a constitutive biological process under physiological conditions, as well as a toxic gain‐of‐function process in dementia.

**RESEARCH IN CONTEXT**

1. **Systematic review**: The authors report the updates and advances in tau research presented at the Tau 2024 Global Conference spanning tau biology, genetics, phenotypes, biomarkers, and therapeutics.
2. **Interpretation**: Tau may serve different functions in different cell types and subcellular locations, under varying circumstances in different diseases, and therefore may participate in a wide range of pathways and mechanisms that can contribute to cell dysfunction and loss.
3. **Future directions**: important aims moving forward will be to identify these mechanisms and pathways and to establish their relative importance in increasingly informative disease‐specific models.

Evans and colleagues conducted a study in which they used human stem cell‐derived neurons to address questions about how different forms of tau (monomeric and aggregated) might enter neurons.^15^ The team discovered two mechanisms by which monomeric tau could enter human neurons: one characterized by a rapid dynamin‐dependent process akin to endocytosis and a second distinguished by a slower actin‐dependent process of macropinocytosis. The researchers found that aggregated tau entry was independent of actin polymerization and typically dynamin dependent, similar to endocytosis and distinct from macropinocytosis, the major route by which aggregated tau is known to enter non‐neuronal cells. The team learned that anti‐tau antibodies can impede monomeric tau entry into neurons; however, they we are unable to prevent the internalization of aggregated tau, which can carry antibody with it into neurons. These data suggest that tau entry into human neurons is a physiological process and, therefore, not exclusively disease‐specific. They also offer a new perspective on immunotherapy approaches that target extracellular tau but do not distinguish between forms of tau thought to propagate disease and forms of extracellular tau that are found in the healthy brain.^6^ Although clinical trials of anti‐tau antibodies so far have yet to report deleterious effects related to the disruption of interneuronal transfer of non‐pathogenic tau, the potential for detrimental effects must be considered in future studies.

Building on these findings, Livesey and colleagues more recently sought to zero in on the cell biology and cellular mechanisms involved in the entry and processing of extracellular monomeric and aggregated tau by human neurons.^16^ Using whole genome, loss‐of‐function clustered regularly interspaced short palindromic repeats (CRISPR) screens of human induced pluripotent stem cell (iPSC)‐derived excitatory neurons, they attempted to identify genes that regulate the uptake and intracellular processing of extracellular monomeric and aggregated tau (MAPT p.P301S), using a lentiviral library of 100,000 guide RNAs targeting 20,000 protein‐coding genes. For both monomeric and aggregated tau, these researchers found that multiple genes involved in endosomal sorting and in the regulation of receptor recycling were required for tau uptake. The uptake of aggregated tau is dependent on several intracellular vesicular trafficking systems, including those involved in trafficking between organelles and surface protein glycosylation. They also discovered that monomeric tau uptake required the recently identified low‐density lipoprotein receptor LRP1^17^ and the endocytosis regulator LRRK2,^18^ mutations in both of which have been associated with familial Parkinson's disease.^19^ Moreover, they found that the genes involved in tau uptake bore a striking similarity to genes required for viral infection – particularly infection by viruses that enter cells via receptor‐mediated endocytosis.

Livesey and colleagues confirmed that monomeric and aggregated tau enter human neurons by means of overlapping but distinct pathways that are dependent on specific surface receptors and on the endolysosomal‐autophagy system, as well as the Golgi network and other vesicle trafficking pathways. Because the cell biology underlying extracellular tau uptake and processing by neurons is analogous to that of viral entry into cells, the authors suggest that this “**quasi**‐infectious” process be considered during the development of potential therapeutic strategies to prevent disease progression.

### 1.3 Tau in the nucleus, nucleolus, and cytoplasm

Tau protein, which is most abundant in neuronal axons, has been found in a variety of intracellular compartments and extracellular locations throughout the central and peripheral nervous systems.^1^ Different tau isoforms have been detected in both neuronal and non‐neuronal cells in multiple cell compartments, and, beginning in the 1990s, researchers have reported the observation of various isoforms of tau in regions of the nucleus and the nucleolus, in both human and cultured primate cell lines.^20^, ^21^, ^22^, ^23^ Although findings from these studies differ depending on protocols and antibodies used, as well as on stage of cell differentiation, all have pointed toward unanticipated roles of tau in both the nucleus and nucleolus.^22^ More recently, researchers have discovered that tau can translocate to the nucleus under cellular stress,^24^ that nuclear tau tends to be non‐phosphorylated,^25^ and that tau likely plays a role in DNA protection. In addition, some works suggest that tau may modulate nucleocytoplasmic transport^26^ or may be linked to nuclear envelope integrity.^27^ Such findings have further underscored tau's potential role in processes of normal cellular physiology that are *not* associated with microtubules, while raising new questions regarding the functional relevance of tau in the nucleus and nucleolus. Current research goals include determining whether specific tau transcripts or isoforms are likely to predict tau's cellular distribution, whether or how tau's functions might change depending on its localization, and how various alterations in both location and function might play a role in different tauopathies.

In recent years Maina and colleagues have continued the examination of nuclear and nucleolar tau with the aim of answering some of these questions, while further elucidating tau's characteristics under physiological and pathological conditions.^22^ In one series of experiments, the team aimed to determine whether tau localized to the nucleolus in terminally differentiated cells such as human neurons.^28^, ^29^ Using transmission electron microscopy (TEM), immunogold labeling, and undifferentiated neuroblastoma cells (SH‐SY5Y cells, a human cell line), the team was able to confirm that tau localized to the nucleolus in both undifferentiated and differentiated SH‐SY5Y cells, where it associates with TIP5, a protein necessary for heterochromatin stability that also plays a role in the repression of ribosomal DNA (rDNA) transcription. The presence of tau in the nucleolus of differentiated cells – a new finding – disproved earlier assumptions that tau likely did not have a role in the nucleolus after differentiation.^21^ In their ongoing work, the team also confirmed the presence of tau in the nucleus and nucleolus in iPSC‐derived cortical neurons and in human brain samples,^29^ and with immunogold labeling they were able to confirm that tau also co‐localized with TIP5 in the nucleolus in human brain samples.^29^

In an effort to characterize tau's role in the nucleolus, Maina and colleagues explored the consequences of tau depletion in SH‐SY5Y cells. The knockdown of tau resulted in an increase in rDNA transcription and an associated decrease in heterochromatin and DNA methylation, which suggested that under normal physiological conditions tau is involved in silencing of rDNA.^29^ Subsequently the team sought to discover whether tau might behave in a manner similar to other nucleolar proteins in response to stress. Using glutamate to induce cellular stress, the team discovered that nucleolar stress did indeed result in the redistribution of nucleolar non‐phosphorylated tau, in a manner similar to the nucleolar protein fibrillarin.^29^ This finding, together with the non‐phosphorylated state of tau, further supports a physiological role for tau in the nucleolus and suggested to the researchers that tau ought to be considered a canonical nucleolar protein.

In a separate study, Maina and colleagues examined phosphorylation as a stress response after exposure to beta amyloid peptide (Aβ).^28^ The incubation of differentiated human neuroblastoma (SH‐SY5Y) cells with Aβ42 oligomers resulted in subtle oxidative stress and nucleolar stress, initially without causing DNA damage or cell death. The presence of exogenous Aβ oligomers resulted in altered nuclear tau levels, as well as phosphorylation, and an altered distribution of nucleolar tau. The team observed that these markers of cellular dysfunction tend to worsen over time and cause a reduction in ribosomal RNA synthesis and processing, a reduced global level of newly synthesized RNA, and reduced protein synthesis. These findings provide evidence for the involvement of Aβ42 in alterations in nucleolar tau in cultured cells and in a corresponding dysfunction of protein synthesis machinery, which has been associated with mild cognitive impairment (MCI) and early stages of AD. In ongoing research, Maina and colleagues are aiming to obtain further details regarding subtle relationships that exist among cellular stress, the phosphorylation of tau, and tau's role in nucleolar functions in the human brain.

Accumulating evidence suggests that pathogenic forms of tau in the cytoplasm can also negatively affect nuclear architecture via their effects on the actin cytoskeleton^30^ and microtubules.^31^

Human brain tissue from patients with AD^26^, ^30^, ^32^ and frontotemporal dementia (FTD) due to the *MAPT IVS10+16* mutation^31^ features invaginations of the nuclear envelope that harbor disease‐associated phosphotau, in line with electron microscopy‐based analyses in 2006 that reported “nuclear contour irregularity” in AD.^33^ Filamentous actin was found to be enriched in nuclear envelope invaginations in human AD brain tissue, and studies in tau transgenic *Drosophila* indicated that tau‐induced overstabilization of the actin cytoskeleton is a mechanistic driver of nuclear envelope invagination.^30^ Studies in iPSC‐derived neurons from patients with FTD carrying various *MAPT* mutations indicate that tau‐induced microtubule dysregulation also contributes to nuclear pleomorphism.^31^

### 1.4 Tau and mitochondrial function in amyotrophic lateral sclerosis

Recent studies have begun to establish links between tau pathology and amyotrophic lateral sclerosis (ALS) pathogenesis in both sporadic and familial cases of ALS, a fatal neurodegenerative disease that affects both cortical and spinal motor neurons.^19^ Although several genes have been implicated in the pathogenesis of ALS, including *SOD1*, *TDP43*, *C9ORF72*, and *FUS*,^19^ mutations in these genes account for a small percentage of all cases. ALS has been associated with a significant increase in total tau as well as cytoplasmic inclusions of hyperphosphorylated tau (T175, T217, S208/210, S212, S396, and S404) in the *post mortem* motor cortex and spinal cord of ALS patients.^34^ Alterations in tau and the ratio of p‐tau to tau also have been reported in the cerebrospinal fluid (CSF) of individuals with ALS.^35^ A number of studies have shown tau‐induced alterations in cellular processes in ALS such as excitotoxicity, mitochondrial dysfunction, synapse loss, and nucleocytoplasmic transport,^36^ which suggests that alterations in tau might be responsible for these molecular events in ALS. In mice, the overexpression of 3R tau specifically in hilar astrocytes of the dentate gyrus has been shown to alter mitochondrial dynamics and function.^37^

With the aim of further exploring tau's role in ALS, Petrozziello and colleagues recently designed a study to investigate the potential role of tau in mitochondrial dysfunction.^38^ Previous studies had suggested that mitochondrial dysfunction was a key pathogenic event in the disease, and studies of AD *post mortem* brain and animal models have suggested a link between alterations in mitochondrial function and interactions between hyperphosphorylated tau and dynamin‐related protein 1 (DRP1) – the GTPase involved in mitochondrial fission. Tau is required for the trafficking of mitochondria across axons to synapses,^39^ which sustains the high energy needs of neuronal cells. Hyperphosphorylation of epitopes on tau impairs this process, disrupts mitochondrial localization, and contributes to axonal dysfunction and synapse loss in AD.^39^ Petrozziello and colleagues sought to determine whether hyperphosphorylated tau may lead to mitochondrial fragmentation and dysfunction in ALS and whether reducing tau may provide a novel approach to treatment.

The team investigated *post mortem* motor cortex samples from 47 people with ALS and 25 controls (including five with a *C9ORF72* expansion and one with an *SOD1* mutation) and were able to report for the first time that pTau‐S396 is mislocalized to synapses in *post mortem* motor cortex across ALS subtypes. The researchers also added synaptoneurosomes that were isolated from ALS brain samples to SH‐SY5Y cells and discovered that treatment with ALS synaptoneurosomes (SNs), enriched in pTau‐S396, increased oxidative stress, induced mitochondrial fragmentation, and altered mitochondrial connectivity in vitro. Because pTau‐S396 had previously been shown to interact with GTPase DRP1^40^ and ALS SNs were shown to induce mitochondrial fragmentation in vitro, the team hypothesized that increases in pTau‐S396 might trigger pathological mitochondrial fission in ALS by binding DRP1. They were able to demonstrate that pTau‐S396 did indeed interact with DRP1 and that DRP1 accumulated in SNs across ALS subtypes, leading to increases in mitochondrial fragmentation in ALS. The team concluded their study by using QC‐01‐175, a selective tau degrader to reduce tau levels, and found that this treatment prevented ALS SN‐induced mitochondrial fragmentation and oxidative stress in vitro.

This study of a large cohort of human *post mortem* mCTX suggests that hyperphosphorylated tau at S396 may indeed underlie mitochondrial fragmentation in ALS by interacting with the pro‐fission GTPase DRP1. The data from the study represent sufficient groundwork for assessing QC‐01‐175 as a novel potential therapeutic strategy for improving mitochondrial morphology and function and, in turn, possible motor neuron survival in ALS.

### 1.5 Genetics

Among the highlights of Tau 2020 were presentations of research that aimed at identifying new gene variants that may play a role in the progression of various tauopathies, as well as potentially protective variants.^1^ Several presentations at Tau 2022 revealed recent advances in the examination of the complexity of human *MAPT* mutations with the goal of refining disease modeling and ultimately enhancing efforts to develop novel tau‐targeting therapeutics.

### 1.6 Functional effects of MAPT splicing and MAPT haplotypes

A detailed understanding of *MAPT* splicing and how it is regulated will likely be a prerequisite for accurately determining the etiology of neurodegenerative diseases in which disease‐specific tau isoforms accumulate in characteristic pathologic inclusions. It is well established that *MAPT* is a neuronally expressed gene comprising 16 exons and that the alternative splicing of *MAPT* exons 2, 3, and 10 results in the expression of six different isoforms in the human brain.^1^ Tauopathies often are categorized by the presence of tau aggregates containing either 3R or 4R microtubule‐binding domain repeats (determined by the inclusion/exclusion of exon 10). More than a decade ago, studies indicated that in several primary tauopathies the regulation of *MAPT* splicing is altered and that misspliced isoforms are differentially incorporated into neurofibrillary tangles (NFTs) and pathogenic inclusions.^41^, ^42^ Although the alternative splicing of *MAPT* exon 10 in healthy and diseased brains has been well characterized, until recently no studies had examined the regulation of exons 2 and 3, and thus the contribution of N‐terminal tau isoforms to primary tauopathies was unexplored.

Recently, Bowles and colleagues observed the coordinated splicing of *MAPT* exons 2 and 10 using a correlational screen for candidate splicing factors and RNA binding proteins in human brain tissue.^43^ The team found that the expression of exon 2 splicing regulators involved in exon 2 inclusion are differentially disrupted in PSP and AD brains, resulting in the accumulation of 1N4R isoforms in PSP and 0N isoforms in AD (temporal cortex). They also identified the presence of different N‐terminal isoforms of tau in NFTs, dystrophic neurites, and tufted astrocytes, which suggests that differential N‐terminal splicing plays a role in the development of disparate tau neuropathologies. The researchers concluded that N‐terminal splicing and combinatorial regulation with exon 10 inclusion/exclusion is likely to be fundamental to the understanding of tauopathies. They proposed that differences in splicing of the *MAPT* N‐terminus that exist between AD and PSP result in the expression of isoforms with different aggregation properties and subcellular localizations, which in turn help to explain the distinct neuropathological phenotypes of each disease. They suggested that investigation of the role of N‐terminal splicing in other primary tauopathies associated with different pathologies – such as Pick's disease, primary age‐related tauopathy (PART), and chronic traumatic encephalopathy (CTE) – would help determine whether such diverse disorders also exhibit loss of *MAPT* exon 2 and 10 splicing coordination. The authors further concluded that it is unlikely that exon 10 splicing alone underlies and regulates disease pathogenesis and tau neuropathology in either AD or PSP; instead, it is likely that the combined expression of specific N‐ and C‐terminal *MAPT* isoforms plays a role in the development of each tauopathy.

At Tau 2022, Bowles also outlined early efforts to determine the functional effects of the two *MAPT* haplotypes, H1 and H2. These collaborative efforts, in association with the Center Without Walls program for research on tau funded by the National Institute of Neurological Disorders and Stroke (NINDS), aim in part to ascertain exactly how and why the H1 haplotype is associated with numerous different neurodegenerative diseases with unique clinical phenotypes. Bowles and other researchers have begun to conduct various omics analyses to determine the functional effects of both haplotypes – in individuals with European ancestry and African ancestry – to determine how differences between H1 and H2 are moderated and exactly which cell types are affected.

### 1.7 Tauopathy‐specific neuroimmune responses

A growing focus on the role of microglia and neuroimmune responses in AD and other tauopathies has pointed to a need for biological models that can help to elucidate the complex microglial responses to diseases of the human brain and how they may vary in different clinical dementia syndromes and during various stages of disease. Microglia play a critical role in coordinating the neural‐immune response resulting from injury and are known to contribute to neuronal dysfunction in multiple ways.^44^, ^45^, ^46^ However, recent studies suggest that microglial responses in neurodegeneration are more varied than previously suspected – for example, single‐cell genomic studies have begun to reveal substantial heterogeneity among disease‐associated microglial states and their trajectories.^47^, ^48^, ^49^ A detailed understanding of the genetic and regulatory drivers of neuroimmune processes and how these contribute to neurodegeneration would be of great value, in part because such information would likely eventually enable disease‐specific selection of immunotherapeutic options.

To understand how neural‐immune‐associated genes and pathways contribute to neurodegenerative disease pathophysiology, Rexach and colleagues conducted systematic functional genomic analyses of microglia and bulk tissue from mouse and human models of AD, FTD, and PSP.^50^ An earlier bulk tissue RNA sequencing (RNAseq) study by the same team revealed that downregulated microglial gene expression trajectories were likely obscured by a general disease‐related upregulation of microglia.^51^, ^52^ Accordingly, the team designed a study that would reveal both upregulated and downregulated signaling pathways within microglia, to enable more precise identification of stage‐ and pathology‐associated microglial states. To achieve this, the researchers integrated cell‐type‐specific, microglial gene expression data from different stages of disease with bulk tissue transcriptomes to enable the identification of disease‐relevant, cell‐specific signaling networks.

This study by Rexach and colleagues involved a systematic, integrative analysis of microglial transcriptomic changes that were linked with neurodegeneration‐associated pathways at the tissue level. They were able to assign disease genes to distinct microglial co‐expression modules that are related to progressive stages of neurodegeneration in genetically diverse mouse models and in the human brain. They found that the common genetic risk factors that contribute to AD, Pick's disease, and PSP involve temporally and biologically distinct microglia‐associated neuroimmune modules that converge on viral responses as a common causal factor. Using multiple data types, integrated across species and human diseases, including chemical genomics experiments, the team demonstrated that early microglia disease response involves a “tension” between immune suppressors and immune activators. Their data and analyses support a model in which neuroimmune signaling in tauopathies is dominated by viral response pathways. The pathways initially involve microglial neuroimmune suppression, driven by type I interferon (IFN) and double‐stranded RNA (dsRNA), followed by the activation of type II IFN during the phase of disease characterized by frank neurodegeneration.

The team discovered complex, disease‐specific microglial trajectories comprising distinct signaling and neuroimmune states in each of the three tauopathies and accordingly determined that tau‐associated dementia syndromes *do* differ with regard to neuroimmune mechanisms. Among the disorder‐distinct neuroimmune responses that they identified were a glial‐immune gene co‐expression module suppressed specifically in PSP (in regions of PSP brain, genes that protect against NK cells were suppressed), distinct lymphocyte profiles in the brain, and tauopathy‐specific regulation of master immune genes (within the HLA locus).

The data obtained by this team were in line with recent findings that IFN‐driven microglial immunosuppression in aging may also contribute to age‐related susceptibility to neurodegeneration.^53^ Moreover, their observation that AD, FTD, and PSP susceptibility genes converge on viral response pathways is consistent with the proposal that the microglial type I IFN response may influence early disease progression, including the propagation of tau pathology.^54^, ^55^ The analyses suggest that a combination of early immune suppression and delayed viral response, rather than immune activation alone, may contribute to disease progression and promote chronic inflammation as tauopathies progress into the clinical phase. The authors concluded that future functional and mechanistic studies will be needed to test and extend their model, which should have significant implications for the development and timing of therapeutic interventions targeting the neuroimmune response.

### 1.8 JADE 1 in tauopathy

In 2014, a new tauopathy, PART, was recognized and subsequently used to describe a pathology that had been commonly observed in the brains of elderly individuals.^56^ A neurodegenerative pathology, PART was determined to have features that were distinct from but also overlapped with AD. While brains with PART are characterized by NFTs that are identical to those that characterize AD, they are distinguished by an absence of amyloid beta (Aβ) plaques. In individuals with PART, symptoms typically range from normal to amnestic cognitive changes but typically do not include profound cognitive impairment.^56^ The need for new terminology became apparent after researchers agreed that clinical/pathologic descriptions such as “tangle‐only dementia” and “tangle‐predominant senile dementia” were imprecise for this age‐related tauopathy, which was almost universally detectable at autopsy among elderly individuals but difficult or impossible to identify in living individuals. Although the new nomenclature helped to raise awareness of this extremely common pathologic entity, PART has continued to be a subject of debate and the focus of questions regarding its ambiguous clinical identity.

Recently, Farrell and colleagues designed a study to find genetic evidence that might be used to clarify the controversial relationship between the neuropathologically similar PART and AD.^57^ Although the pathogenesis of PART was not known, evidence to date suggested an association with genes that promote tau pathology and/or protect from Aβ toxicity. The research team performed an autopsy‐based, neuropathology‐based, genome‐wide association study (GWAS) using the largest cohort (*n* = 647) of *post mortem* brain tissues from aged individuals that lacked Aβ neuritic plaque pathology and met all other criteria for PART.^57^ A primary goal was to identify factors that were independently associated with PART. Using Braak NFT stage as a quantitative trait, the team observed significant associations with candidate loci associated with AD (*SLC24A4*, *MS4A6A*, *HS3ST1*) and PSP (*MAPT* and *EIF2AK3*). Further analysis revealed a novel significant association with a single nucleotide polymorphism on chromosome 4 (rs56405341) in a locus containing three genes, including *JADE1*, which was significantly upregulated in tangle‐bearing neurons. Immunohistochemical studies using antisera that targeted JADE1 protein revealed its localization in tau aggregates in brains with four microtubule‐binding domain repeat (4R) isoforms and mixed 3R/4R, but not with 3R exclusively. Co‐immunoprecipitation in *post mortem* human PART brain tissue revealed a specific binding of JADE1 protein to 4R tau lacking N‐terminal inserts (0N4R).

Farrell and colleagues confirmed that while the genetics of PART overlap to some degree with sporadic late‐onset AD, individuals with PART have a higher *APOE* 𝜀2 allele frequency, which distinguishes PART from AD both neuropathologically and genetically, and a lower frequency of the *APOE ε4* allele, as demonstrated in previous studies in independent cohorts. Their findings reinforced prior evidence that PART occurs independently of *APOE ε4*. The team's immunohistochemical studies indicated that JADE1 may be involved in 4R and mixed 3R/4R tauopathies; they observed immunopositivity not only in PART tangles, but also in tangles of tauopathies with aggregates that contained 4R tau and in mixed tauopathies with aggregates that contained both 3R and 4R tau. However, the team was surprised to discover an absence of staining in Pick's disease.

The findings from this study indicate that PART has a genetic architecture that partly overlaps with AD and other tauopathies and suggest a novel role for JADE1 (which interacts with 0N4R tau and is protective in vivo) as a modifier of neurofibrillary degeneration. The authors note that additional studies in experimental models will be necessary to validate their findings and improve our understanding of the genetics of PART, which in turn could lead to new opportunities for rationally designed tau therapeutics.

### 1.9 Phenotypes

Numerous current tau research studies are aiming to develop an integrative view of the intricate links between genotypes and phenotypes with the aim of using this information to construct in detail and define the clinical evolution and diversity of the tauopathies.

### 1.10 Phenotypes in a Colombian cohort

At Tau 2022, Diana Matallana presented intriguing data obtained from inhabitants of Aranzazu, a small town in Colombia, that demonstrated some of the challenges of studying and gathering information from an admixed population. Aranzazu is one of many regional areas in Colombia whose population originated from a historical tri‐continental admixture of diverse indigenous peoples, Spanish invaders, and enslaved Africans, who have been geographically separated for tens of thousands of years.^58^ After these populations experienced significant mortality from a number of deadly infectious diseases – including smallpox, influenza, encephalitis, tuberculosis, cholera, typhus, and meningitis – a “bottleneck” resulted in survivors who were geographically dispersed into relatively isolated small admixed populations. A proportionally higher frequency of rare variants derived from the ancestral populations has been reported in a study of the genomes from 900 Colombian individuals with AD, frontotemporal lobar degeneration (FTLD)‐motor neuron disease, early‐onset dementia not otherwise specified, and healthy participants, with 21 pathogenic variants in AD‐FTLD‐related genes and PSEN1 representing the majority.^58^ These populations currently are a focus of genomic studies that aim to understand the disease burden of underrepresented populations, ascertain and/or assess the transferability of risk scores from European cohorts, and characterize the unique genotype–phenotype relationships that exist in these cohorts.

Just as rare variants in genes from these populations provide novel perspectives on the range of associated clinical phenotypes and point to potential underlying molecular pathways for various tauopathies, the observation of unique clinical phenotypes and their familial aggregation suggest the likely presence of rare genetic variants that have yet to be detected.

### 1.11 The presymptomatic stages in FTD/MAPT mutation carriers

Approximately one third of cases of FTD, a heterogenous neurodegenerative disorder, are caused by genetic variants, among which variants in *GRN*, *MAPT*, and *C9orf72* are the most common. Although much is known about the clinical features of these genetic forms of FTD, few studies conducted before 2020 provided very large sample sizes from which to draw information about age at symptom onset and disease duration. In a large international retrospective cohort study, Moore, Rohrer, and colleagues analyzed ages at symptom onset and death and disease duration, examining both the effect of mutation type and family membership.^59^ Their study showed that both age at symptom onset and at death among people with genetic FTD was directly influenced by genetic grouping. Among individuals with *MAPT* mutations, these characteristics were directly affected by both the specific mutation carried by an individual and by family membership. The authors concluded that estimation of age at onset would be an important factor in future presymptomatic therapeutic trials for all three genetic groups, while data from other family members likely would be of value only for individuals with *MAPT* mutations.

Subsequently, members of the FTD Prevention Initiative (FPI), led by Jonathan Rohrer at University College London in the United Kingdom and Adam Boxer at University of California San Francisco (UCSF) in the USA, brought together genetic FTD cohorts from across Europe, North America, South America, Australasia, and Asia to further investigate phenotypic differences among individuals with FTD. A primary goal has been to gain greater insight into the presymptomatic stages of genetic FTD – the accumulation of progressive molecular and cellular changes in the nervous system that occur before the onset of dementia – which might offer opportunities to delay or even prevent neurodegeneration by means of early therapeutic intervention with targeted molecular therapies.

Wilke and colleagues designed a study of a large multicenter cohort of genetic FTD mutation carriers (the Genetic FTD Initiative, or GENFI cohort, *n* = 444), with the goal of providing a biomarker‐based stratification of participants, as well as documentation of the biomarker cascade during the presymptomatic phase of FTD.^60^ The research group obtained longitudinal assessments of serum levels of neurofilament light (NfL) and phosphorylated neurofilament heavy (pNfH). Participants in the study comprised 91 symptomatic and 179 presymptomatic subjects with variants in the FTD genes *C9orf72, GRN*, or *MAPT*, as well as 174 mutation‐negative within‐family controls. The researchers detected a biomarker cascade, such that increase in NfL preceded hypothetical clinical onset by 15 years and concurred with brain atrophy onset, while increases in pNfH began to occur closer to clinical onset. The conversion stage was marked by increased NfL but normal pNfH levels, while both biomarkers were elevated at the symptomatic stage. The finding that intra‐individual rates of change were increased for NfL at the conversion stage and for pNfH at the symptomatic stage pointed to their respective potential as stage‐dependent dynamic biomarkers within the biomarker cascade. Increased NfL levels and NfL rates of change enabled the identification of presymptomatic individuals who were converting to symptomatic disease and also permitted estimations of proximity to onset. Finally, exploratory analysis of the three genetic subgroups of mutation carriers suggested a NfL increase at the presymptomatic stage, followed by a pNfH increase with the onset of symptoms for *C9orf72* and *GRN*, but a slower NfL increase and apparent absence of pNfH increase for *MAPT*.

Wilke and colleagues demonstrated that blood NfL and pNfH values could permit dynamic stage‐dependent stratification of individuals with FTD and might serve as treatment‐response biomarkers in presymptomatic FTD that help to demarcate the conversion stage. The team's proposed biomarker cascade is expected to help facilitate a biomarker‐based precision‐medicine approach to genetic FTD.

### 1.12 Mapping disease in presymptomatic MAPT mutation carriers

Neuroimaging studies of *MAPT* mutation carriers have pointed to a number of well‐established patterns of disease in the brain. For example, behavioral variant FTD (bvFTD) due to *MAPT* mutations is, like sporadic bvFTD, characterized by degeneration in the anterior cingulate cortex, insula, striatum, and the amygdala; in addition, bvFTD‐*MAPT* also is known to more prominently target the mesial temporal lobe (in particular, the hippocampus) – a region that is less likely to exhibit atrophy in sporadic bvFTD.^61^, ^62^ However, reliable information about the timing of changes in *brain volume* among *MAPT* mutation carriers – particularly during the presymptomatic phases of disease – has been difficult to obtain. Many studies have provided conflicting or unclear information regarding gray and white matter volume trajectories in presymptomatic individuals, and most studies have failed to account for individual anatomic variation.

To gather information about gray and white matter differences in individuals with these mutations, Chu, Lee, and colleagues studied a multisite cohort of 65 *MAPT* mutation carriers (22 symptomatic and 43 presymptomatic) from whom they obtained structural magnetic resonance imaging (MRI) scans with a voxel‐wise method that enabled detection of gray and white matter differences in individual carriers.^63^ They hypothesized that regions of low gray or white matter volume in presymptomatic *MAPT* mutation carriers would resemble atrophy patterns seen in symptomatic carriers, and they also anticipated that a subset of presymptomatic carriers – individuals likely closer to symptom onset – would have gray and white matter volumes lower than expected for their age. A primary goal was to determine whether different *MAPT* mutation subtypes affected distinct neuroanatomical regions.

Clinical syndromes varied among the symptomatic participants in the study, such that 18 had bvFTD, two had an amnestic dementia syndrome, one had Parkinson's disease, and one had MCI. The investigators performed voxel‐based morphometry on T1 images and assessed brain volumetrics by clinical subgroup, age, and mutation subtype. These assessments revealed that symptomatic carriers exhibited gray matter atrophy in regions that included the bilateral frontotemporal cortex, insula, and striatum and exhibited white matter atrophy in regions of the bilateral corpus callosum and uncinate fasciculus. The study also revealed that approximately 20% of presymptomatic carriers had low gray matter volumes in voxels within the bilateral hippocampus, amygdala, and lateral temporal cortex, and within these regions, low gray matter volumes emerged in a subset of presymptomatic carriers as early as their thirties. Among presymptomatic carriers, low white matter volumes were only infrequently observed.

The research team found that a subset of presymptomatic carriers in their thirties had low mesial temporal volumes – a finding that was consistent with an earlier study in which gray matter trajectories revealed low hippocampal and amygdala volumes arising 15 years before estimated symptom onset.^64^ Their data indicated that presymptomatic carriers exhibited low volumes within canonical regions that are targeted in *MAPT* mutations. Their findings suggest that a subset of presymptomatic participants in the study may be undergoing incipient neurodegeneration and that more carriers will likely follow suit as symptom onset approaches. An intriguing finding was that the frequency of low mesial temporal lobe volumes appeared to outpace that of other regions, suggesting that the mesial temporal lobe is targeted early in *MAPT* mutation carriers and with increasing frequency, both with age and during the symptomatic phase of disease.

### 1.13 LATE versus FTLD‐TDP nomenclature

A presentation by Julie Schneider at Tau 2022 explored recent efforts to introduce a change in nomenclature to the AD/AD and related dementias (ADRD) field, with the aim of addressing important phenotypic variability among older individuals with a progressive amnestic syndrome and autopsy evidence of transactive response DNA binding protein of 43 kD (TDP‐43) pathology with or without AD pathology. TDP‐43 pathology was first reported in 2006 as a primary component of ubiquitinated inclusions in autopsy‐confirmed cases of FTLD that were negative for tau immunoreactivity but positive for ubiquitin and is now known as FTLD‐TDP.^65^, ^66^ The introduction of the new nomenclature for clinical disease related to TDP‐43 pathology – limbic‐predominant age‐related TDP‐43 encephalopathy, or LATE,^67^ and LATE neuropathologic changes, or LATE‐NC – followed more than a decade of published papers in AD/ADRD research addressing the relationship of TDP‐43 with our without AD pathology and memory loss (without clinical diagnosis of FTD) or TDP pathology specifically with AD neuropathologic changes.^68^, ^69^, ^70^, ^71^

Schneider and colleagues published a paper in which they defined LATE‐NC as a stereotypical TDP‐43 proteinopathy in older adults, with or without coexisting hippocampal sclerosis pathology.^67^ The co‐authors presented LATE‐NC as a common TDP‐43 proteinopathy, associated with an amnestic dementia syndrome that has mimicked AD‐type dementia (progressive amnestic syndrome) in retrospective autopsy studies. They noted that LATE is distinguished from FTLD TDP‐43 pathology partly by its epidemiology (LATE primarily affects older individuals) and partly by the relatively restricted neuroanatomical distribution of the TDP‐43 proteinopathy. They found that among community‐based autopsy cohorts, approximately 25% of brains had LATE‐NC associated with discernible cognitive impairment and that individuals with LATE‐NC typically had comorbid brain pathologies, which often include Aβ plaques and tauopathy.

Because people at the greatest risk for LATE‐NC are among the “oldest‐old” – a rapidly growing demographic group in many countries – the authors noted that LATE has a growing and yet underrecognized impact on public health. To stimulate research and promote awareness of this particular path to dementia, the co‐authors convened a working group to develop diagnostic criteria for LATE. The group reported consensus‐based recommendations, including guidelines for the diagnosis and staging of LATE‐NC. Recommendations for routine autopsy workup of LATE‐NC included an anatomically based preliminary staging scheme, involving TDP‐43 immunohistochemistry on tissue from three brain areas (amygdala, hippocampus, and middle frontal gyrus), to address a hierarchical pattern of brain involvement. The group noted that although LATE‐NC appears to affect the medial temporal lobe structures preferentially, it also affects other areas of the brain, and that neuroimaging studies of individuals with LATE‐NC in some cases demonstrated atrophy throughout the temporal lobe, with lesser but consistent involvement of the frontal cortex, and other regions of the brain.

To date, genetic studies have indicated five genes with risk alleles for LATE‐NC: *GRN, TMEM106B*, *ABCC9, KCNMB2*, and *APOE*. The working group has noted that, although discovery of these genetic risk variants indicate that LATE shares pathogenic mechanisms with both FTLD and AD, disease‐specific underlying disease mechanisms also have been apparent for LATE‐NC. While recognizing that significant gaps remain in our knowledge of LATE, research focused on LATE – including research involving in vitro and animal models – will be critical to ensuring appropriate advances in the prevention, diagnosis, and treatment of the disease. The group has emphasized the urgent need for diagnostic tools, such as biofluid or neuroimaging biomarkers, for the ante mortem detection of LATE, not only to strengthen studies that seek to further define its risk factors, natural history, and clinical features, but also to enhance subject recruitment for eventual targeted therapies in clinical trials.

### 1.14 Phenotypic variability of PSP

PSP is a 4R tauopathy that has been classified as belonging to the broader category of FTLD‐tau disorders.^72^ The presence of NFTs and threads in subcortical nuclei as well as the presence of tufted astrocytes are among the primary criteria for the neuropathological diagnosis of PSP, and oligodendroglial coiled bodies and diffuse cytoplasmic immunoreactivity in neurons may also be observed.^73^ Although cases of PSP were initially roughly categorized as typical, atypical, or combined pathologies,^74^ evidence of biochemical differences among cases of PSP as well as differences in the amount of pathology emerged and gradually led to the recognition of different PSP phenotypes. By 2017, clinicians and researchers recognized distinct clinical subtypes that included PSP‐Richardson Syndrome (RS), PSP with corticobasal syndrome (PSP‐CBS), with progressive gait freezing (PSP‐PGF), with predominant ocular motor dysfunction (PSP‐OM), with predominant postural instability (PSP‐PI), with predominant frontal presentation (PSP‐F), and with predominant speech and language disorder (PSP‐SL).^75^

Although sequential tau distribution patterns have been recognized for tau pathologies such as Pick's disease, argyrophilic grain disease, and astrocytic tau pathologies, as well as for other proteinopathies,^76^ the development of a scoring or staging system that incorporated sequential distribution patterns in PSP presented a challenge because of the range of its tau cytopathologies and clinical phenotypes. To address the question of whether sequential distribution patterns could be recognized and incorporated into a classification of subtypes for PSP pathology, Kovacs and colleagues designed a large international study of PSP in *post mortem* brains involving the evaluation of heat maps and distribution patterns of neuronal, astroglial, and oligodendroglial tau pathologies, as well as their combinations in different clinical subtypes of PSP.^77^

Using conditional probability and logistic regression to model the sequential distribution of tau pathologies across different brain regions, the researchers found that tau pathology uniformly manifested in the neurons of the pallido‐nigro‐luysian axis in different clinical subtypes. They were able to distinguish clinical subtypes of PSP not only according to total tau load but also according to cell‐type (neuronal vs glial) specific patterns of vulnerability across brain regions, which suggested distinct dynamics or circuit‐specific segregation of propagating tau pathologies. For RS they were able to recognize six sequential steps of involvement in brain regions based on observed combinations of cellular tau pathologies. The co‐authors noted that these sequential steps implied six stages of practical neuropathological diagnosis, comprising evaluation of the subthalamic nucleus, the globus pallidus, the striatum, the cerebellum with dentate nucleus, and the frontal and occipital cortices. The authors recommended further application of this system to other clinical subtypes, which could be categorized as caudal (cerebellum/dentate nucleus) or rostral (cortical) predominant or as comprising both types of pattern.

Efforts to define cell‐specific stages of tau pathology are expected to improve the ability to identify preclinical or early‐stage cases of PSP and will also likely advance our understanding of early pathogenic events in PSP. Although current clinical diagnostic criteria will continue to inform clinical subtype‐specific dynamics of disease‐propagation, more research will be necessary to improve our ability to predict PSP pathology, and prospective studies of cohorts with PSP will be necessary for the identification of valuable disease‐specific biomarkers.

## 2 BIOMARKERS

### 2.1 Tau‐PET imaging in individuals with cognitive impairment

Positron emission tomography (PET) radiotracers for visualizing Aβ plaques and tau‐containing NFTs in vivo enable diagnostic and prognostic evaluation of individuals and facilitate the investigation of disease mechanisms.^78^, ^79^ While research has shown that amyloid PET is more accurate for diagnosing AD in the earliest stages, recent studies suggest that tau‐PET, a newer technique, may be more advantageous for determining the disease stage and predicting disease progression. In one study of patients in early symptomatic stages of AD, La Joie and colleagues compared Aβ PET and tau‐PET with regard to their ability to predict brain atrophy during a 15‐month period. Using quantitative analysis, the team found that the global intensity of the tau‐PET signal, but not the Aβ‐PET signal, predicted the rate of subsequent atrophy from baseline, independent of cortical thickness at baseline.^80^ Further investigations demonstrated that the specific distribution of the tau‐PET signal was a strong indicator of the topography of future atrophy (at the single patient level) and that the relationship between baseline tau‐PET and subsequent atrophy tended to be stronger in younger patients.^80^ The data gathered by this team supported current disease models that characterize tau pathology as a major driver of local neurodegeneration, and their findings underscored the value of tau‐PET as a precision medicine tool that might be used in the design of future clinical trials to help predict individual patient progression.

The development of AD therapeutics requires a better understanding of the fine details of tau pathophysiology. One particular goal is to better understand individual patterns of tau pathology that do not fit well into the Braak staging system.^81^ Although the Braak staging system,^82^, ^83^ which describes a progression from the transentorhinal cortex to the medial and basal temporal lobes and subsequently into neocortical associative regions, before progression into the unimodal sensory and motor cortex, provides reliable information at the population level, it does not explain systematic variability at the individual level. In a study designed to examine and better characterize individual variability, Vogel and colleagues analyzed tau‐PET scans from 1612 individuals.^84^ The research team was able to identify four distinct spatiotemporal trajectories of tau pathology, which ranged in prevalence from 18% to 33% among the patient scans analyzed. The team discovered posterior and lateral temporal patterns representative of atypical clinical variants of AD and also replicated previously described limbic‐predominant and medial temporal lobe‐sparing patterns. The “subtypes” identified during the study remained stable during longitudinal follow‐up and were replicated in an analysis of a separate sample in which a different radiotracer was used. The subtypes presented with distinct demographic and cognitive profiles and characteristic longitudinal outcomes. Based on their findings, the researchers concluded that variation in tau pathology was common and systematic and may necessitate a revisiting of the staging of tau pathology.

### 2.2 Creating a p‐tau217 clock

When selecting participants for AD prevention trials, it is important to accurately predict a cognitively normal individual's age of symptom onset. Indeed, in clinical trials that have tested treatments for autosomal dominant AD, the ability to make such predictions has been shown to increase the power of a trial while decreasing its costs.^85^ Because the results of anti‐amyloid trials increasingly suggest that disease‐modifying treatments will likely be most effective during a particular stage of disease, the accurate prediction of symptom onset would likely help to accelerate the development of preventive treatments for AD.

A number of studies to date have suggested that the rate of amyloid accumulation in the human brain tends to be slow and highly variable at very low levels of amyloid burden.^86^, ^87^, ^88^ However, after a particular threshold of amyloid burden is crossed, the rate of amyloid accumulation tends to increase and become relatively consistent across individuals, allowing reliable estimations of the timing of amyloid accumulation with various mathematical methods.^89^, ^96^, ^97^ In one study, Schindler and colleagues aimed to predict when cognitively normal individuals with brain amyloidosis would develop symptoms of AD by means of amyloid PET with Pittsburgh compound B (PiB).^90^ After evaluating amyloid accumulation in 236 individuals who underwent more than one amyloid PET scan, the investigators transformed the mean cortical standardized uptake value ratio (SUVR) into a timescale using longitudinal data. They identified a tipping point in amyloid accumulation at a low level of amyloid burden (SUVR 1.2), after which nearly all individuals accumulated amyloid at a relatively consistent rate, eventually reaching a high level of amyloid burden (SUVR 3.0). The average time between levels of amyloid burden was used to estimate the age at which an individual reached SUVR 1.2, and longitudinal clinical diagnoses for 180 individuals were aligned by the estimated age at SUVR 1.2. In the 22 individuals who progressed from cognitively normal to a typical AD dementia syndrome, the estimated age at which an individual reached SUVR 1.2 predicted the age at symptom onset. Schindler and colleagues were therefore able to conclude that the age at symptom onset in sporadic AD is strongly correlated with the age at which an individual reaches a tipping point in amyloid accumulation.

More recently, Schindler and colleagues have investigated whether changes in fluid biomarkers could also be used to identify a tipping point in amyloid aggregation that could, in turn, be used to align longitudinal clinical data across individuals and enable prediction of the onset of AD symptoms in cognitively normal individuals. Schindler's team analyzed CSF data from 385 participants in longitudinal studies at the Knight Alzheimer's Disease Research Center, generated by Nicolas Barthélemy and Randall Bateman. They discovered that after reaching a tipping point, concentrations of CSF p‐tau217 increased relatively consistently over a period of approximately 30 years – a length of time considerably longer than the 18 years captured by the PiB PET amyloid clock. The investigators found that an individual's p‐tau217 levels during this 30‐year period could be used to estimate the chronological age at which the person reached the tipping point in CSF p‐tau217 concentrations. Further, the age at the tipping point was correlated with the age at onset of dementia. Overall, this analysis suggests that fluid biomarkers may be useful in estimating an individual's age at symptom onset.

### 2.3 Advances in blood‐based biomarkers

Well‐established pathophysiological hallmarks of AD (amyloid, tau, and neurodegeneration) currently are detectable in CSF or by imaging, such as amyloid‐PET and tau‐PET.^91^, ^92^ There is an urgent need to develop cost‐effective biomarkers that are less invasive and that can be serially measured. At Tau 2022, Oskar Hansson and Charlotte Teunissen presented a number of promising advances in the identification and validation of blood‐based biomarkers that suggested the likelihood of their successful implementation in clinical trials and in clinical practice in the near future.

Tau has more than 70 posttranslational modification sites, including more than 40 phosphorylation sites and several truncated forms, and currently a number of different p‐tau forms are not only measurable in both CSF and plasma but also quite informative.^93^ The concentrations of plasma tau phosphorylated at three particular sites (pTau181, pTau217, or pTau231) are significantly increased in individuals with clinically diagnosed AD compared with both cognitively unimpaired controls and individuals with non‐AD dementias.^94^ Independent studies strongly suggest that plasma p‐tau181, for example, reflects AD‐specific neuropathology because p‐tau181 is elevated in individuals with AD compared with those who have non‐AD dementias, including other tauopathies.^94^, ^95^, ^96^ In a prospective cohort study of both cognitively impaired and unimpaired individuals, both baseline and longitudinal changes in plasma p‐tau181 were associated with widespread tau aggregation 6 years later.^97^ Moreover, p‐tau181 has been used to differentiate participants with amyloid pathology across different clinical stages, particularly in areas of the brain affected by AD.^94^

Among some of the most promising developments in the arena of p‐tau biomarkers have been those involving the tau variant phosphorylated at Thr217 (p‐tau217). Plasma levels of p‐tau217 have been shown to have a strong association with tau pathology in the brain, particularly in the presence of Aβ plaques in *post mortem* tissue,^98^ a finding that is in line with imaging studies that have demonstrated strong correlations between plasma p‐tau217 and tau‐PET scans in individuals with AD but not in those with non‐AD tauopathies.^96^ Research findings to date suggest that the phosphorylation of tau at Thr217 might represent a subtle and unique aspect of tau pathology that is different from the other isoforms, although differences could also be related to assay and reagent specificities. Wennström/Hansson and colleagues recently designed a study to investigate the rise in plasma p‐tau217 in AD, with the aim of identifying potential cellular/pathological mechanisms that contribute to the rise.^99^ They explored the cellular localization of p‐tau217, compared with that of five other p‐tau variants (p‐tau181, 231, 202, 202/205, and 369/404), in the Cornu Ammonis 1 (CA1) of the hippocampus of AD patients. They also analyzed the presence of p‐tau217 in four different areas of the brain (CA1, entorhinal cortex [EC], inferior temporal gyrus [ITG], and superior frontal gyrus [SFG]) of neuropathologically diagnosed individuals. They subsequently aimed to determine whether the p‐tau217 load in these areas of the brain correlated with p‐tau217 concentrations in ante mortem plasma.

Using immunostaining techniques to assess *post mortem* AD brain tissue, the research team showed that p‐tau217 was found in NFTs and neuropil threads that are also positive for p‐tau181, 202, 202/205, 231, and 369/404. The p‐tau217 variant, but not the other five, was also prominently observed in vesicles positive for markers of granulovacuolar degeneration bodies and multivesicular bodies. The team found significantly higher p‐tau217 area fraction in individuals with a strong likelihood of AD in four different areas of the brain (EC, ITG, and SFG), compared with individuals who had PART or other non‐AD tauopathies. The p‐tau217 area fraction correlated strongly with total Aβ and NFT brain load, based on analyses of the entire group, and the mean p‐tau217 area fraction was significantly correlated with p‐tau217 concentrations in ante mortem plasma – specifically in individuals with amyloid plaques (though not in those without amyloid plaques). These findings provided new information about the differences in cellular localization among different p‐tau variants and suggested that plasma levels of p‐tau217 reflect an accumulation of p‐tau217 in the presence of Aβ plaque load.

As blood‐based biomarkers of tau pathology, such as p‐tau 217, approach clinical use, it will be essential to determine factors that may affect the concentrations of these markers to accurately interpret results.^100^ Such information will be especially important for the development of reference ranges.^94^ Initial blood biomarker studies typically are conducted in well‐characterized populations, but it will be necessary to understand the factors that affect values in diverse population‐based and community‐based cohorts. Factors such as age, sex, comorbidities, medication, lifestyle factors, and genetic variation can affect the clinical interpretation of blood biomarkers. The examination of blood‐based biomarkers in diverse communities will be important for understanding racial, ethnic, and geographical differences, which have already been shown to affect AD CSF biomarker and Aß‐PET values.^101^, ^102^

To address these and other critical issues, Teunissen and colleagues have recommended an adaptation and graphical representation of the Geneva roadmap,^103^ which describes a five‐phase framework for biomarker development. They note that the communication of biomarker results to potential users, including clinicians and patients, should be a key aspect of phase 5 of their roadmap implementation, to facilitate advanced care planning and enable patients to make informed choices for the future.^94^, ^104^

## 3 THERAPEUTICS AND CLINICAL TRIALS

### 3.1 Benefits of tau reduction

Most tau research to date has focused on associations between the accumulation of intraneuronal tau aggregates and the development of various neurodegenerative disorders. However, a growing number of studies are suggesting that tau also may play an indirect or “enabling” role in some tau‐related diseases and disorders. An analogy drawn from the therapeutic area of infectious disease illustrates this concept: it is well established that HIV‐1 uses the C‐C chemokine receptor type 5 (CCR5) to enter T lymphocytes and that the receptor therefore plays a key role in the pathogenesis of HIV/AIDS; indeed the genetic loss of this receptor has been shown to prevent the development of HIV disease.^3^, ^105^ Although CCR5 is not an active effector or mediator of HIV immunopathogenesis, it serves as an enabler of the infection process. In a similar manner, there is growing evidence that the physiological presence and/or functions of tau may “enable” neural dysfunction and behavioral abnormalities caused by other pathogenic drivers in some disorders, including autism, depression, epilepsy, and stroke.^3^, ^106^

The potential enabling role of tau has been suggested in various studies in which the reduction of physiological, non‐aggregated wild‐type (WT) tau in the brain has prevented or reduced neural network and behavioral dysfunctions in relevant experimental disease models.^3^, ^106^, ^107^, ^108^ Although the mechanisms by which such dysfunctions are ameliorated are unknown, tau reduction may alter neurons in ways that reduce the occurrence of epilepsy‐promoting processes such as excitation/inhibition (E/I) imbalance, hyperexcitability, and hypersynchronization, which are believed to contribute to cognitive impairments and behavioral alterations in a range of disease models.^108^, ^109^, ^110^

Chang and colleagues recently investigated the effects of tau ablation on the activity of excitatory neurons by recording spontaneous action potential (sAP) firing of pyramidal cells (PCs) in layer 5 of the somatosensory cortex (L5) in acute cortical slices from WT and tau‐deficient (*Mapt−*/−) mice. The investigators selected this brain region as an area of focus because of its involvement in epilepsy and AD^111^ and because the morphological differences between PCs and interneurons in this region facilitate efforts to distinguish these entities on recordings. Global genetic ablation of tau in these mice reduced the action potential (AP) firing and E/I ratio of pyramidal cells in acute cortical slices without affecting the excitability of these cells. Chang and colleagues were able to demonstrate that tau ablation is indeed capable of reducing excitatory inputs to inhibitory neurons, increasing the excitability of these cells, and structurally altering their axon initial segments (AIS). They further showed that in primary neuronal cultures subjected to prolonged overstimulation, tau ablation could diminish the homeostatic response of AIS in inhibitory neurons, promote inhibition, and suppress the occurrence of hypersynchrony.

The manner in which global tau ablation affects excitatory pyramidal cells and inhibitory interneurons and in turn reduces the E/I ratio of neural networks may represent a cellular/network mechanism by which therapeutic tau reduction might address diseases with aberrant increases in E/I ratios. To date, tau reduction has been shown to prevent or reduce epileptiform activity, behavioral abnormalities, and/or premature death in models of AD, autism, depression, epilepsy, and stroke.^3^ A tau‐targeting antisense oligonucleotide (ASO) developed for this purpose^112^ is currently undergoing testing in a clinical trial for early AD (ClinicalTrials.gov identifier NCT03186989). Other ASO approaches are also under investigation.^113^

### 3.2 Exploring the possibilities of genome surgery

The Clelland laboratory at UCSF has been exploring the potential development of cures for dementia and other neurodegenerative diseases using CRISPR gene editing to address monogenic causes of these diseases. The team is currently developing novel CRISPR‐based therapeutic gene‐editing technologies to test whether gene editing could be used to safely reverse cellular pathology with genetic causes in patient‐derived cells. They have begun by investigating the use of genome “surgery” for FTD and ALS, which are linked by a shared genetic cause – a heterozygous hexanucleotide (GGGGCC) repeat expansion in a single allele of the *C9orf72* gene.

Clelland and colleagues recently evaluated three approaches to editing the mutant *C9orf72* gene (excision of the repeat region, excision of the mutant allele, and excision of regulatory region exon 1A) to determine the relative viability of each approach in correcting pathology in neurons derived from patient iPSCs. These three strategies were selected for their therapeutic potential because they did not involve template‐based gene correction (which is known to be inefficient in postmitotic neurons) and because they minimized off‐target editing. Each strategy depended on Cas9's ability to cut DNA, a technique considered closest to “clinical prime time.” The researchers found that all three approaches normalized RNA abnormalities and TDP‐43 pathology, but only excision of the repeat region and the mutant allele excision completely eliminated pathologic dipeptide repeats.

The findings from this initial gene‐editing study will allow the team to advance to further preclinical testing of repeat expansion excision and allele‐specific excision, to determine which of these two approaches is likely to be more efficient and precise in postmitotic neurons as well as in vivo models. Their current findings suggest that excision of the repeat expansion is less efficient in diseased iPSCs.

Their preliminary findings also warrant further investigation across patient lines with various repeat lengths and, ultimately, in differentiated patient‐derived neurons. The team is eager to develop and adopt delivery technologies for additional preclinical testing with the aim of advancing their techniques for use in clinical trials. Their robust editing and outcome measurement tools have begun to lay the groundwork for investigating gene‐editing approaches to monogenic disease in human iPSCs and in derived cell types and should be applicable to any monogenic disease – particularly other repeat expansion disorders. Their initial study demonstrated the usefulness and reliability of single‐molecule sequencing to characterize large repeat expansions and verify their excision; accordingly, the team recommends that this approach become the gold standard for future studies of repeat expansion diseases.

Reproduction of the team's findings across other patient lines will be important – especially in those with different repeat expansion sizes – and a detailed understanding of the organization of each target gene locus and expression will be vital to ensure that unexpected potential side effects are adequately detected/avoided. So far this work sheds light on the complex regulation of the *C9orf72* gene and suggests that because of sense and antisense transcription, silencing a single regulatory region may not reverse all pathology. The team's work has resulted in a roadmap for evaluating CRISPR gene correction using patient iPSCs – research that could eventually lead to a single curative intervention.

### 3.3 Tau as biomarker enrichment tool in clinical trials

Rapid developments in research focused on tau‐PET imaging in recent years have led to the consideration of tau‐PET for the diagnosis of neurodegenerative diseases.^114^ Evidence to date indicates that the currently available tau tracers consistently bind to the paired helical filaments of AD‐type tau, with less reliable binding in non‐AD tauopathies, such that tau‐PET is considered a promising tool for the differential diagnosis of various tauopathies and the identification of atypical AD phenotypes that can be difficult to diagnose.^114^ During the next few years studies that address the clinical impact of tau imaging – for example, whether it might be capable of resolving diagnostic ambiguity^115^ and those that compare tau imaging with both established and newer plasma biomarkers – will be particularly important.

While the role of tau‐PET as a biomarker continues to evolve, it already has proven useful in the selection of patients for a clinical trial.^116^ In a phase 2 trial of donanemab in patients with early symptomatic AD, screening procedures included PET with injection of 18F‐flortaucipir, MRI, and PET with injection of 18F‐florbetapir. Patients were required to have flortaucipir PET scans that showed evidence of pathologic tau deposition but with quantitative tau levels below a specific upper threshold to address the concern that anti‐amyloid treatments likely have limited efficacy in advanced disease (characterized by the presence of extensive tau pathology). Flortaucipir PET scans in this trial were quantitatively evaluated to estimate tau SUVRs according to published methods and were visually evaluated to ensure detection of a tau deposition pattern consistent with AD. Patients with a SUVR of more than 1.46 and patients with a SUVR of less than 1.10, as well as those with a deposition pattern not consistent with AD, were excluded from the trial (except for patients with a SUVR of less than 1.10 who *did* have a topographic deposition pattern consistent with advanced AD, who were included). The protocol was such that patients were required to meet all eligibility criteria at the first visit (except for undergoing MRI) before undergoing screening with florbetapir PET. The sequence of screening procedures and the flortaucipir PET criteria ensured that only a small percentage (0.9%) of patients assessed for eligibility who met the flortaucipir PET criteria did not meet the florbetapir PET criterion (amyloid SUVR ≥1.17, equivalent to 37 centiloids). In addition to excluding patients with the highest tau levels, who are hypothesized to have disease more resistant to anti‐amyloid treatments, the flortaucipir PET screening criteria also may have narrowed the range of underlying pathologic features and in turn decreased variation in clinical decline.

As tau‐PET is costly and involves the injection of a radioactive tracer, the field has welcomed the recent arrival of blood‐based biomarkers. In a number of recent studies, these blood assays have been used alongside tau‐PET imaging to evaluate their diagnostic performance versus imaging. An analysis of donanemab phase 2 trial data, for example, investigated the value of plasma p‐tau217 (tau phosphorylated at threonine 217) for determining response to treatment.^117^ The analyses showed that treatment with donanemab resulted in an early reduction of p‐tau217 values, and a significant reduction (*p* < 0.01) was observed at the 3‐month timepoint compared to placebo. Investigators found that a decrease in p‐tau217 values correlated significantly with amyloid change at all time points, at 24 weeks, and at 76 weeks. Investigators concluded that these data supported the amyloid cascade hypothesis and suggested that amyloid‐related tauopathy can be altered along with donanemab's impact on plaque clearance. They further concluded that the p‐tau 217 data suggested early and profound amyloid clearance could translate into clinical benefit for patients.

Encouraging findings regarding the value of p‐tau217 led to an adaptation of the phase 3 trial named TRAILBLAZER‐ALZ2 that likewise incorporated the use of a plasma biomarker. For this phase 3 trial of people with early AD, scientists incorporated a plasma p‐tau181 assay as a prescreening tool for enrollment. Plasma p‐tau181 was measured for a subset of 752 potential enrollees before they underwent amyloid‐ and tau‐PET scans, and 3619 other potential enrollees underwent PET scans without p‐tau 181 prescreening. Among the 752, p‐tau181 predicted the presence of both amyloid and tau pathology. Sixty‐three percent of individuals with elevated blood p‐tau181 subsequently were found to have both plaques and tangles on PET; among those who underwent tau‐PET scan without p‐tau181 prescreening, only 37% were found to have both types of pathology. These findings suggested that in future trials, plasma p‐tau prescreening might help reduce costs by eliminating PET imaging and making trials more accessible to individuals without access to PET centers.

### 3.4 Opportunities for early intervention

Although abnormal levels of Aβ are now typically among inclusion criteria for AD clinical trials,^118^, ^119^ and results from anti‐amyloid trials have suggested that it will be important to recruit individuals at earlier stages of Aβ accumulation, less attention has been given to levels of tau accumulation in clinical trial recruitment. A greater understanding of the prognostic value and anatomical distribution of tau accumulation in cognitively unimpaired individuals can play a role in informing future trial design.

In a recently published study, Strikwerda‐Brown and colleagues aimed to determine the clinical value of NIA‐AA research criteria in the assessment of older individuals without cognitive impairment who are at near‐term risk of developing symptomatic AD.^120^ The study involved assessments of 128 individuals from the Pre‐symptomatic Evaluation of Experimental or Novel Treatments for Alzheimer Disease (PREVENT‐AD) cohort, 153 from the Harvard Aging Brain Study (HABS), 48 from the Australian Imaging, Biomarker & Lifestyle (AIBL) study, and 251 from the Knight Alzheimer Disease Research Center (ADRC). All participants underwent at least 1 Aβ and tau‐PET scan, were cognitively unimpaired at the time of PET scanning, and underwent at least 12 months of clinical follow‐up. Across cohorts, 33% to 83% of amyloid and tau‐positive (A+T+) participants progressed to MCI during follow‐up, compared with less than 20% of participants in other biomarker groups. Progression further increased to 43% to 100% among A+T+(N+) individuals. Many A+T+ participants who did not progress to MCI nevertheless exhibited longitudinal cognitive decline, though cognitive trajectories in groups with less tau remained stable. The investigators were able to confirm the clinical prognostic value of NIA‐AA research criteria in all four independent cohorts and demonstrated that most A+T+(N+) older individuals without cognitive impairment developed AD symptoms within 2 to 3 years.

In another recent study, Insel and colleagues examined regional rates and spatial patterns of tau accumulation in cognitively unimpaired older adults across multiple cohorts of cognitively unimpaired individuals to determine how these patterns related to amyloid burden, with the goal of designing optimal tau endpoints for clinical trials.^121^ Using three large cohorts of cognitively unimpaired older adults, from the Anti‐Amyloid Treatment in Asymptomatic Alzheimer's (A4) and its companion study, Longitudinal Evaluation of Amyloid Risk and Neurodegeneration, the Alzheimer's Disease Neuroimaging Initiative, and the Harvard Aging Brain Study, the investigators identified regions of the brain with high rates of tau accumulation and attempted to estimate how these rates might evolve over a continuous spectrum of amyloid deposition, beginning at baseline. The inferior temporal cortex, fusiform gyrus, and middle temporal cortex were the most noteworthy regions of accumulation, with the largest effect sizes in both longitudinal cohorts, when considered individually. Insel and colleagues found that in both longitudinal studies the inferior temporal cortex, almost exclusively, was the optimal region of interest for participants with mildly elevated Aβ levels. For participants with highly elevated baseline Aβ levels, a combination of regions provided optimal information, with composite weights of 53% for the inferior temporal cortex, 31% for the amygdala, and 16% for the fusiform region. The investigators noted that, although previous findings indicated that the EC was the first site to show abnormal levels of tau with age, these abnormal levels are unlikely to be the result of a high rate of short‐term accumulation. They concluded that while the EC plays a central role in the early appearance of tau, it may be that the inferior temporal cortex is the critical region for rapid tau accumulation in preclinical AD, a finding that may play a significant role in improving the selection of participants for preventive trials.

## 4 RECOGNITION

The Rainwater Prize for Outstanding Innovation in Neurodegenerative Disease Research recognizes an investigator whose work is considered a significant contribution to our understanding of tau‐related diseases. Researchers from all countries and research institutional affiliations may be nominated, and awardees are chosen by a committee of international scientific leaders from a wide range of fields and backgrounds. At Tau 2022, the 2022 Rainwater Prize for Outstanding Innovation in Neurodegenerative Disease Research was awarded to Dr. Alison Goate, the Jean C. and James W. Crystal Professor and Chair of Genetics at Icahn School of Medicine at Mount Sinai, who has been a leader in the genetics of tauopathies for more than 30 years. Her early work focused on Mendelian forms of dementia and led to the identification of mutations in *APP*, *PSEN1*, and *MAPT* as causes of AD and FTD. Her groundbreaking discoveries paved the way for mechanistic studies and therapeutic development. More recently, Dr. Goate has been a leader in the use of 2D‐ and 3D‐organoid cultures to better understand how variants influence dementia risk and has provided valuable insight into the molecular changes produced by these genetic risk factors.

The Rainwater Prize for Innovative Early Career Investigator is intended to reward the outstanding achievements of a scientist who is either first author or senior author on published neurodegenerative disease research findings. The prize aims to support exceptionally productive scientists who are in the early formative stages of their academic career (within 10 years of primary investigator appointment) and who plan to make a long‐term career commitment to research aligned with the mission areas of the Rainwater Charitable Foundation. At Tau 2022, Martin Kampmann, PhD, Associate Professor for Neurodegenerative Diseases at the UCSF, was awarded the 2022 Rainwater Prize for Innovative Early Career Investigator. Dr. Kampmann and his lab conducted the first genome‐wide CRISPR screens in human neurons to systematically identify genes that control neuronal survival. Kampmann also played a key role in providing the first molecular description of neurons that are particularly vulnerable in AD. Dr. Kampmann's work was recognized for using cutting‐edge technology to discover novel biological insights.

## 5 SUMMARY

As tau researchers continue to apply more precise tools and innovative experimental approaches to the goal of refining our knowledge of tau functions, the development and spread of tau pathology, and the states of disease progression that are characteristic of each of the tauopathies, unanticipated findings are regularly calling into question earlier, fundamental assumptions about tau. Indeed, a number of experts at Tau 2022 presented novel findings that may point to the need for a shift in thinking and/or a reframing of current research questions. Deeper examinations of the non‐canonical, physiologic functions of tau, for example, have led to findings with important implications for anti‐tau/immunotherapeutic approaches to treatment – particularly those that currently target extracellular tau without distinguishing between tau forms associated with disease and those that are present in the healthy brain. Newer findings also have revealed or confirmed the existence of new categories of tauopathies – in some cases, requiring a change in nomenclature – as well as a more detailed understanding of disease staging, which has increasingly suggested that the success of a treatment may depend in large part upon its timing. The limitations of animal models and in vitro research also are becoming increasingly apparent, as research continues to reveal that the means by which tau aggregation and related pathological processes take place in human cells during disease most likely involve numerous complex and diverse pathological processes that are difficult or impossible to duplicate.

Many current lines of investigation in the field of tau today are placing a greater emphasis than ever on the value of collaboration. While most of the earliest tau research took place within the context of AD, for example, presentations at Tau 2022 underscored tau's role in a wide range of disorders, ranging from classic tauopathies such as PSP and CBD to an expanding list of conditions not classically categorized as tauopathies, such as Parkinson's disease, Down syndrome, autism, and epilepsy. As research exploring the primary functions of tau in both disease and physiology moves forward, improved mechanisms for sharing discoveries regarding each of the individual disorders, including critical genetic discoveries, will likely help to guide research on related tauopathies and steer experts/colleagues from paths unlikely to be fruitful. Important collaborative goals will include the elucidation and mapping of the signaling pathways that are relevant to a range of biological and pathological processes in each of the tauopathies, as well as their regulation, with the aim of arriving at increasingly informative disease‐specific models that might in turn lead to effective therapeutic strategies.

### 5.1 Glossary

Taber's Cyclopedic Medical Dictionary [Internet]. In: Venes DD, editors. Taber's Medical Dictionary. F.A. Davis Company; 2021. [cited 2023 May 23]^122^

### 5.2 Amyotrophic lateral sclerosis (ALS)

A motor neuron disease characterized by the degeneration of anterior horn cells of the spinal cord, the motor cranial nerve nuclei, and the corticospinal tracts. This disorder limits one's ability to use the upper and lower extremities and/or to speak and swallow.

### 5.3 Corticobasal degeneration (CBD)

A neurological disorder in which brain cells atrophy and die in the basal ganglia and the cortex of the brain. The disease produces symptoms similar to those found in Parkinson's disease but does not respond to parkinsonian medications.

### 5.4 Chronic traumatic encephalopathy (CTE)

Dementia associated with repeated concussions and/or other brain injuries.

### 5.5 CRISPR

CRISPR is an acronym for “clustered regularly interspaced short palindromic repeats.” It refers to an enzyme that can be guided to cut and edit specific sequences of DNA. It is a biochemical tool originally identified in bacteria, which use it for self‐defense against viral and other infections. It is used in genetic engineering (genome editing) to identify and remove disease‐causing DNA sequences.

### 5.6 Endocytosis

A method of ingesting a foreign substance by a cell. The cell membrane invaginates to form a space for the material, and the opening subsequently closes to trap the material inside the cell.

### 5.7 Isoform

One of two or more proteins coded independently by different genes, which have identical or nearly identical structures and functions.

### 5.8 Induced pluripotent stem cell (iPSC)

A cell derived from the body that has been reprogrammed to behave like an embryonic stem cell. Such cells are able to differentiate into cells that could regenerate and repair many different kinds of damaged or diseased tissues.

### 5.9 Macropinocytosis

The process by which cells absorb or ingest nutrients and fluid. An invaginating portion of the cell membrane encircles the nutrient, encloses it in a membrane‐bound sac, and digests the contents of the sac.

### 5.10 Microtubule

An elongated (200 to 300 Å) hollow or tubular structure present in cells. Microtubules are important in helping certain cells maintain their rigidity, in converting chemical energy into work, and in providing a means of transporting substances in different directions within a cell. They increase in number during mitosis.

### 5.11 Nucleolus

A spherical structure in the nucleus of a cell made of DNA, RNA, and protein that is the site of synthesis of ribosomal RNA (rRNA); a cell may have more than one. Embryonic and malignant cells actively synthesize rRNA and have larger nucleoli.

### 5.12 Progressive supranuclear palsy (PSP)

A chronic progressive neurodegenerative disorder in which features of symmetrical Parkinson's disease are combined with dementia, falls, impaired gait, and vertical gaze paresis. Features that distinguish PSP from Parkinson's disease include the relative preservation of the sense of smell in PSP and differences in response to levodopa. The disorder is caused by damage to cells in the frontal lobes, the basal ganglia, the cerebellum, and the brainstem. Brain MRI may demonstrate loss of brainstem parenchyma (the “hummingbird sign”).

## CONFLICT OF INTEREST STATEMENT

Claire Sexton has nothing to disclose. G. Bitan has nothing to disclose. K. R. Bowles has nothing to disclose. M. Brys reports patents planned, issued, or pending; stock or stock options; other financial or non‐financial interests from Eli Lilly & Co. L. Buée reports grants or contracts from T‐PEP AA and RCF, USA; ANR, France; consulting fees: Aptah Bio, USA; Beckman Coulter, USA; support for attending meetings: ADPD, AAIC; patents: EP22306999.8 Methods for decreasing therapeutic acquired resistance to chemotherapy and/or radiotherapy, December 2022; EP 21306903.2 Methods for improving the efficacy of HDAC inhibitor therapy and predicting the response to treatment with HDAC inhibitor, December 2021. M. Bukar – Maina reports grants or contracts from the Alzheimer's Association Research Fellowship; University of Sussex; support for attending meetings: Alzheimer's Research UK South Coast Network, University of Sussex Neuroscience, Alzheimer's Association Research Fellowship; leadership or fiduciary role: Society of Neuroscientists of Africa, Yobe State Government, TReND in Africa. L. B. serves on the Wellcome Trust LMIC Advisory Board. C. D. Clelland reports grants or contracts from BrightFocus Foundation Fellowship, Alzheimer's Association Clinician Scientist Fellowship, NIH/NINDS K08‐NS112330KO8, Carol and Gene Ludwig Award for Early Career Research, Larry H. Hillblom Fellowship, Wolfen Family Foundation, and Wozniak Family gifts. A. D. Cohen reports grants or contracts: R01 AG052446, R01 AG072641, P30 AG066468, P01 AG025204‐16, U19 AG068054, R01 AG063525, R37 AG023651, RF1 AG025516, RF1 AG052525, U19 AG024904‐01, AG078109‐01, P50 MH130957‐01, R01 AG075992‐01, U01 NS100610‐06; receipt of AV1451 precursor from Avid. J. F. Crary reports grants or contracts: NIH grants R01AG054008, R01NS095252, RF1AG060961, R01NS086736, R01AG062348, RF1MH128969, P30AG066514, R01AG063819, R01NS116006, U54NS115266; support for attending meetings: Rainwater Charitable Foundation/Tau Consortium. J. L. Dage reports grants or contracts: NIH/NIA institution, Roche institution, Clinical and Translational Science Institute; consulting fees: Genotix Biotechnologies Inc., Gates Ventures, Karuna Therapeutics, AlzPath Inc., Cognito, AbbVie, Eisai; payment or honoraria: Eli Lilly and Company; patents: patents filed relating to assays, methods, reagents, and/or compositions of matter used in this work – assigned to Eli Lilly and Co.; leadership or fiduciary role: ADC Biomarker Steering Committee; Vice Chair ISTAART BBB PIA; stock or stock options: Eli Lilly and Co. minor shareholder, AlzPath, Monument Biosciences, Genotix Biotechnologies Inc.; receipt of equipment, materials, drugs, medical writing, gifts, or other services: Roche Diagnostics, ADx Neurosciences, Eli Lilly and Co. K. Diaz has nothing to disclose. B. Frost reports grants or contracts: 1 R01 AG078964, 1 R01 AG058778‐01, 1 RF1 NS112391‐01, 1 R01 AG074289, 1 R01 AG062475‐01A1, UTHSCSA Pepper Center, Center for Biomedical Neurosciences Pilot Grant, William and Ella Owens Medical Research Foundation, Transposon Therapeutics, Rainwater Foundation/Tau Consortium, MD Anderson Neurodegeneration Consortium; consulting fees: MD Anderson Neurodegeneration Consortium; paid travel: 2023, Tau Consortium and 2022, Tau Consortium from Rainwater Foundation; 2023, Neurodegeneration Consortium, 2022, Neurodegeneration Consortium from MD Anderson; 2023, Glenn Workshop from AFAR. Leadership or fiduciary role: co‐organizer, Tau 2024 Conference; associate editor, Progress in Neurobiology. L. Gan reports grants or contracts: NIA/NIH: R01AG072758, NIA/NIH: R01AG074541, NIA/NIH: 1R01AG077899, Rainwater Foundation; JPB Foundation all funding to Weill Cornell. Consulting fees: Ono Pharma USA; honoraria: 17th Wisconsin Stem Cell Symposium; 8th Annual Neuroimmunology and Glia Group (NGG) retreat; Mayo Clinic Department of Neuroscience Virtual Seminar; University of Pittsburg, Aging Research Seminar Series; University of Arizona School of Pharmacy. Travel and accommodation support: DZNE Lecture Series in Bonn, Bonn, Germany; DZNE/Synergy seminar series, Munich, Germany; GRC on Neurobiology of Brain Disorders: Understanding disease mechanisms and developing novel therapeutics for neurodegeneration, Castelldefels, Spain; Cold Spring Harbor Meeting: Neurodegenerative Diseases and Biology; AD/PD 2023, Presymposium; Keystone Symposia on Neuroimmune Interactions and Neurodegeneration; Alzheimer's Association International Conference; GRC Neuroimmune Communication in Health and Disease; 8th Annual Neuroimmunology and Glia Group (NGG) retreat; Spring Brain Conference; BIG Symposium, Washington University in St. Louis, School of Medicine; FBRI Workshop on Alzheimer's Disease. Patents: US patent application: “cGas Inhibitors and Uses Thereof” 63/309,894 ‐ one of the inventors. Equity holder: Aeton Therapeutics. A.M. Goate reports grants or contracts: JPB Foundation; Rainwater Charitable Foundation; Cure Alzheimer's fund paid to institution. Royalties or licenses: Taconic Industries, Athena Diagnostics; consulting fees: Genentech, Muna Therapeutics. Payment or honoraria: Biogen, Genentech. Support for attending meetings: Rainwater Charitable Foundation; patents: 6,475,723 Pathogenic tau mutations – Licensed to Taconic Industries; 5,973,133 Mutant S182 genes; 5,877,015 APP770 mutant in AD. Stock or stock options: Cognition Therapeutics, Denali Therapeutics. L. I. Golbe reports royalties or licenses: Rutgers University (PSP Rating Scale, video guide to the PSP rating scale), Rutgers University Press (A Clinician's Guide to Progressive Supranuclear Palsy [2019]). Consulting fees: AI Therapeutics, Amylyx, Aprinoia, Ferrer (Asceneuron), Mitochon, Switch, UCB. Travel support from CurePSP as Chief Clinical Officer and board of directors member. Participation on DSMB or AB for Rossy Centre, University of Toronto; leadership or fiduciary role: CurePSP: Chair, Scientific Advisory Board; Chief Clinical Officer; member, board of directors; member, Executive Committee; President, Dwight Morrow High School Alumni Educational Alliance (Englewood, NJ). O. Hansson reports consulting fees: AC Immune, Amylyx, Alzpath, BioArctic, Biogen, Cerveau, Eisai, Eli Lilly, Fujirebio, Merck, Novartis, Novo Nordisk, Roche, Sanofi, Siemens. Participation on DSMB or AB for: AC Immune, Amylyx, Alzpath, BioArctic, Biogen, Cerveau, Eisai, Eli Lilly, Fujirebio, Merck, Novartis, Novo Nordisk, Roche, Sanofi, Siemens. C. M. Karch reports grants or contracts: NIH, Rainwater Charitable Organization. H. C. Kolb report patents: inventor on patent pending for Janssen plasma p217+tau assay; stock from Johnson & Johnson, parent company of Janssen R&D. H. C. Kolb is an employee of Janssen R&D, receiving salary from said institution. R. La Joie reports grants or contracts: Alzheimer's Association, NIH/NIA, US Department of Defense. Support for attending meetings from Alzheimer's Association. S. E. Lee reports grants or contracts: NIH‐NIA, Bluefield Project; royalties or licenses: UptoDate; support for attending meetings: Tau Consortium. D. Matallana reports grants or contracts: NIH and Fogarty International Center (FIC) under Award R01AG057234, an Alzheimer's Association grant (SG‐20‐725707‐ReDLat); support for attending meetings: NIH and FIC under Award R01AG057234. B. L. Miller reports grants or contracts: NIH/Univ. of Wisconsin, Madison 1R01AG070883, NIH/NIA: R35AG072362, P01 AG019724, P30AG062422, R01AG057234, R01AG062562, R01AG062588, Bluefield Project to Cure FTD, UCSF FTD Core P0544014, NIH/NINDS R01 NS050915, NIH CSR R01AG052496. Royalties or licenses: Cambridge University Press, Elsevier, Inc., Guilford Publications, Inc., Johns Hopkins Press, Oxford University Press, Taylor & Francis Group, all payments made to him. Payment or honoraria: Global Summit on Neurodegenerative Diseases, Korean Dementia Society, Massachusetts General Hospital, dementia course, National MS Society, Don Paty Lectureship, Ochsner Neuroscience Institute, Providence Saint Joseph Medical Center, Taipei Medical University, Dementia Center, UC Irvine Institute for Memory Impairments and Neurological Disorders (UCI MIND), University of California, Los Angeles (UCLA) Grand Rounds, University of Texas Center for Brain Health, all payments made to him. Support for attending meetings: Association for Frontotemporal Degeneration (AFTD) Education Symposium, Milken Institute FTD Scientific Retreat, California Institute of the Arts. Participation on a DSMB or advisory board: Arizona Alzheimer's Consortium, Association for Frontotemporal Degeneration, Buck Institute for Research on Aging, Cure ALS, The John Douglas French Alzheimer's Foundation, Fundación Centro de Investigación Enfermedades Neurológicas, Genworth, The Larry L. Hillblom Foundation, Massachusetts General Hospital ADRC, National Institute for Health Research Cambridge Biomedical Research Center and its subunit, the Biomedical Research Unit in Dementia, Stanford University ADRC, University of Southern California P01 Urban Air Pollution and Alzheimer's Disease: Risk, Heterogeneity, and Mechanisms, University of Washington ADRC. Leadership or fiduciary role: Bluefield Project to Cure FTD, Global Brain Health Institute, Institute for Neurodegenerative Diseases, Tau Consortium of the Rainwater Charitable Foundation. C. U. Onyike reports grants or contracts: Alector, Inc., Transposon Therapeutics, Denali Therapeutics; consulting fees: Acadia Pharmaceuticals, Reata Pharmaceuticals, Otsuka Pharmaceutical. Honoraria for lecture: Philadelphia Psychiatric Society, American Academy of Neurology Institute. Leadership or fiduciary role: AFTD Medical Advisory Council, FTD Disorders Scientific Advisory Board, Tau Consortium Scientific Advisory Board, ISFTD Executive Committee, ISTAART FTD PIA Executive Committee. Receipt of drug and materials for clinical trial: Alector Inc., Transposon Therapeutics, Denali Therapeutics. Y. T. Quiroz reports grants or contracts: Alzheimer's Association, Massachusetts General Hospital ECOR, National Institute on Aging; consulting fees from Biogen. J. E. Rexach has nothing to disclose. J. D. Rohrer reports participation on a DSMB or advisory board: Aviado Bio, Arkuda Therapeutics, Prevail Therapeutics, Denali, Wave Life Sciences. A. Rommel reports a mixed stock funds portfolio in retirement accounts – example: Vanguard, Morgan Stanley, technology funds. G. Sadri‐Vakili has nothing to disclose. S. E. Schindler reports grants or contracts: Barnes‐Jewish Hospital Foundation, National Institute on Aging Grant R01AG070941 – funding paid to institution for research. Consulting fees: Eisai; payment or honoraria: University of Wisconsin, St. Luke's Hospital, Houston Methodist Medical Center, University of Washington, University of Indiana. Support for attending meetings: National Institute on Aging Grant R01AG070941. Leadership or fiduciary role: Greater Missouri Alzheimer's Association. Plasma Ab42/Ab40 data was provided to Washington University by C2N Diagnostics at no cost. J. A. Schneider reports receipt of consulting fee from Cerveau Inc. R. A. Sperling reports consulting fees from AC Immune, Acumen, Alnylam, Cytox, Genentech, Janssen, JOMDD, Nervgen, Neuraly, Neurocentria, Oligomerix, Prothena, Renew, Shionogi, Vigil Neuroscience, Ionis, Vaxxinity all paid directly. C. E. Teunissen's research on CET is supported by the European Commission (Marie Curie International Training Network, Grant Agreement 860197 [MIRIADE]), Innovative Medicines Initiatives 3TR (Horizon 2020, Grant 831434), EPND (IMI 2 Joint Undertaking [JU], Grant 101034344) and JPND (bPRIDE), National MS Society (Progressive MS Alliance), Alzheimer's Association, Health Holland, the Dutch Research Council (ZonMW), Alzheimer's Drug Discovery Foundation, The Selfridges Group Foundation, Alzheimer Netherlands. CT is a recipient of ABOARD, which is a public–private partnership (PPP) receiving funding from ZonMW (No. 73305095007) and Health∼Holland, Topsector Life Sciences & Health (PPP allowance, No. LSHM20106). C. E. Teunissen has a collaboration contract with ADx Neurosciences, Quanterix, and Eli Lilly, performed contract research or received grants from AC‐Immune, Axon Neurosciences, BioConnect, Bioorchestra, Brainstorm Therapeutics, Celgene, EIP Pharma, Eisai, Fujirebio, Grifols, Instant Nano Biosensors, Merck, Novo Nordisk, PeopleBio, Roche, Siemens, Toyama, Vivoryon. C. E. Teunissen received payment or honoraria from Roche, Novo Nordisk, Grifols, all paid to her institution. C. E. Teunissen serves on editorial boards of Medidact Neurologie/Springer, Alzheimer Research and Therapy, Neurology: Neuroimmunology & Neuroinflammation. S. C. Weninger reports consulting fees from Denali Therapeutics. SCW is a member of the board of directors of Neumora Therapeutics, Atalanga Therapeutics, Sironax, Aratome, Eikinizo, TargetALS; Chair, board of directors, and CEO of Rugen Therapeutics, holds stock or stock options of Denali Therapeutics, Atalanta Therapeutics, Neumora Therapeutics, Rugen Therapeutics. S. L. Worley reports consulting fees, payment, or honoraria and support for attending meetings from the Alzheimer's Association. H. Zheng has nothing to disclose. M. Carrillo reports, in the past 36 months, participating on a data safety monitoring board or advisory board for US POINTER and holding a role for EASTERSEALS. Author disclosures are available in the Supporting Information.

## Supporting information

Supporting Information
